# Food Safety, a Global Challenge

**DOI:** 10.3390/ijerph13010067

**Published:** 2015-12-22

**Authors:** Mieke Uyttendaele, Eelco Franz, Oliver Schlüter

**Affiliations:** 1 Department of Food Safety and Food Quality, Faculty of Bio-Science Engineering, Ghent University, Coupure Links 653, B-9000 Gent, Belgium; 2 National Institute for Public Health and the Environment (RIVM), Antonie van Leeuwenhoeklaan 9, 3721 MA Bilthoven, The Netherlands; eelco.franz@rivm.nl; 3 Leibniz Institute for Agricultural Engineering Postdam-Bornim e.V., Max-Eyth-Allee 100, 14469 Potsdam, Germany; oschlueter@atb-potsdam.de

## 1. Introduction

To provide more food and make use of precious water and nutrient resources, communities increasingly value sustainable food production. However, this should be done safely to maximize public health gains and environmental benefits. Food safety is being challenged nowadays by the global dimensions of food supply chains, the need for reduction of food waste and efficient use of natural resources such as clean water. Food safety deals with safeguarding the own national food supply chain from the introduction, growth or survival of hazardous microbial and chemical agents. But within a larger international context, borders are fading and surely this is the case for foodstuffs which are an important globally traded commodity. There is great divergence in the degree of organization, infrastructure, teaching capacity across countries and food protection (food quality, food preservation, food safety) needs to be tackled globally. This special issue assembled topics in food safety, with case studies of food safety concerns from various parts of the world, research on risk factors in agricultural production of fresh produce, use of water and water treatment technologies in food production, and outlooks on food safety for vulnerable persons. The main conclusion throughout all papers is that ensuring food safety of the food supply chain is a continuous challenge and needs our attention.

## 2. Safety Aspects of Foods in Low and Middle Income Countries 

Although evidence on foodborne disease is still limited, most of the known burden of foodborne disease in low and middle income countries comes from biological hazards is mentioned by Delia Grace in her review on food safety in low and middle income countries in this Special Issue [1]. Most foodborne disease in these countries is assumed to be the result of consumption of fresh, perishable foods sold in informal markets. Foodborne disease is likely to increase in low and middle income countries as the result of massive increases in the consumption of risky foods such as livestock and fish products and fresh produce. 

**Figure 1:**
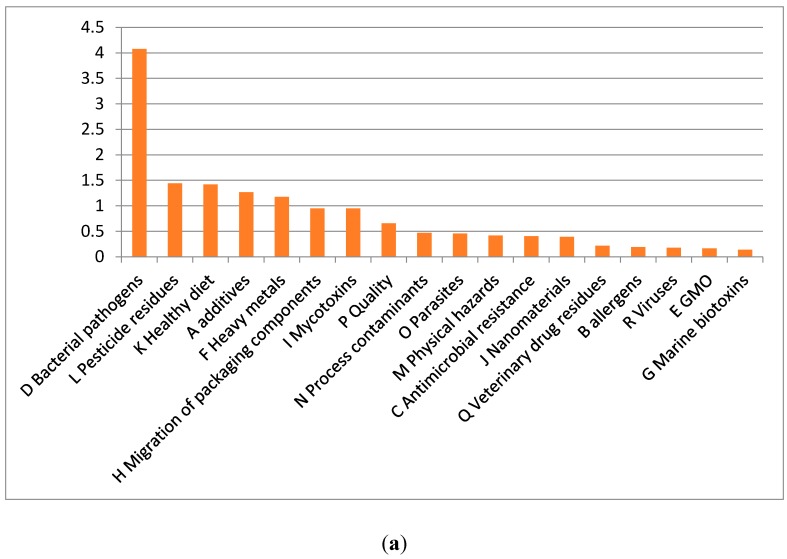

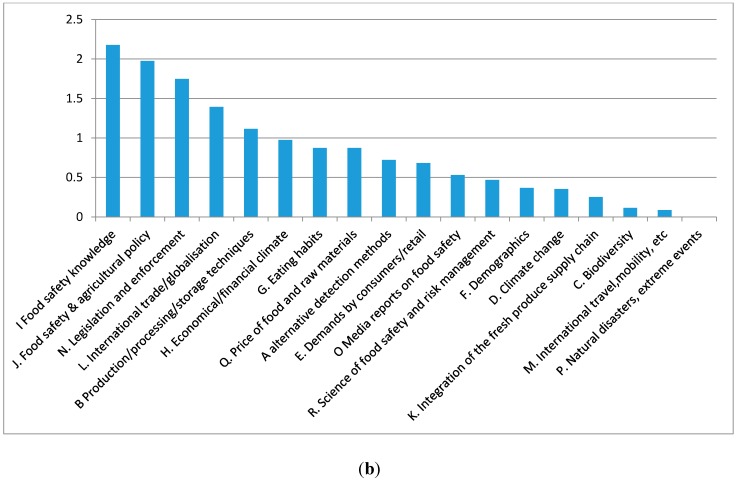
(**a**) main food safety issues of concern and (**b**) main contextual factors perceived to impact food safety (consultation of ITP Food safety Trainees at Ghent University, 2011–2015, *n* = 79 participants). Ranking performed as described by Van Boxstael *et al*. [2] with overall ranking of the items based on equal weighting of the opinions of each participant for allocation of an average importance score between 0 (least important item) and 5 (most important item).

Food safety issues that were expressed by the trainees participating in the Belgian VLIR UOS funded Intensive Training Program in Food Safety, Quality Assurance and Risk assessment (www.itpfoodsafety.UGent.be) at Ghent University also provides insights on global concerns on food safety. Upon their arrival in Ghent, Belgium, trainees were asked to express their opinion on food safety in their country. To facilitate a common starting point for discussion on food safety, the trainees were provided with alphabetically ordered short lists of: (i) food safety issues of potential concern and (ii) contextual factors affecting the safety of the food supply chain as described by Van Boxstael *et al*. [2]. Throughout the years 2009–2015 in total 79 participants coming from 29 countries across the world including Bangladesh, Belgium, Benin, Bolivia, Brazil, Cameroon, Colombia, Cuba, Ethiopia, Ghana, India, Indonesia, Italy, Jordan, Kenya, Nepal, Nigeria, Palestine, Philippines, Rwanda, South-Africa, Sri-Lanka, Sudan, Tanzania, Thailand, Togo, Uganda, Vietnam and Zimbabwe took part in this survey. The main concern in food safety mentioned were bacterial pathogens, followed by pesticide residues and healthy diet (Figure 1a). 

Furthermore, it became clear from the survey that overall the contextual factors mainly impacting on food safety were perceived to be lack of food safety knowledge and need of health, food safety and agricultural policy and appropriate legislation and enforcement by government (Figure 1b).

In many countries milk and dairy production is extremely important for thousands of peasant families and smallholders. It has become part of a strategy of diversification of agricultural activities, risk management and security search. Considering the fact that milk is an excellent environment for the growth of different kinds of microorganisms, microbial hazards are the most important concern within the dairy industry. Still in the case of rural milk production and dairy manufacturing some bottlenecks are identified on the implementations of “best practices” and difficulties are highlighted in implementing food safety regulations by small companies. A study by Akindolire *et al*. [3] in the North-West Province of South Africa showed that raw, bulk and pasteurised milk is potentially contaminated with toxigenic and multi-drug resistant *S. aureus* strains. There is a need to implement appropriate control measures to reduce contamination as well as the spread of virulent *S. aureus* strains and the burden of disease in humans. 

For many countries, fish and fishery productions are an important part of their animal related diet. Fresh aquatic products are vulnerable to contamination with both chemical and microbiological hazards, one of them being shellfish poisons. Zhang *et al*. [4] showed that volatile basic nitrogen can serve as a hygiene index value of live fresh aquatic products and that neurotoxic, amnesic and diarrheic shellfish poisons (NSP, ASP and DSP) are important risk factors for the occurrence of food-borne diarrhea in the population of the Ningbo area in China.

## 3. Pathogenic *Escherichia coli* and *Salmonella*, an Emerging Issue in Fresh Produce

*Escherichia coli* belongs to the normal intestinal microbiota of humans and animals and the majority of them are not harmful. Certain *E. coli* however harbor virulence factors and can cause intestinal and extra-intestinal diseases. Shiga toxin-producing *Escherichia coli* are zoonotic bacteria, and thus often associated with foods of animal origin (especially beef and sheep meat). However, *E. coli* O157:H7 shed in cattle manure can survive for extended periods of time and intervention strategies to control this pathogen at the source are critical as fresh produce crops are often grown in proximity to animal raising operations. Ravva and Korn [5] evaluated whether neem (*Azadirachta indica*), known for its antimicrobial and insecticidal properties, can be used to amend manure to control *E. coli* O157:H7.

Foodborne outbreaks from fruits and vegetables and border rejections of fresh produce due to non-compliance testing are on the rise, generate economic loss and food waste, and lead to a loss of trust and confidence in the safety of fresh produce. A few studies in this Special Issue were focused on safety of fresh produce combining observations of the local situation with sampling and testing of crops and the production environment (soil, water, contact surfaces) to identify risk factors for contamination with enteric bacterial pathogens. Johannessen *et al*. [6] investigated the occurrence of generic *E. coli*, *Campylobacter*, *Salmonella* and pathogenic Shiga-toxin producing *E. coli* (STEC) on strawberries and the strawberries’ production environment. Ceuppens *et al*. [7] assembled the results from a multi-country study and found that generic *E. coli* was a suitable index organism for *Salmonella* and STEC when sampling leafy greens and strawberries at primary production and production environment, but to a lesser extent for *Campylobacter*. Fruit and vegetables being a commodity often grown in open fields with intensive use of water, the latter study also showed that temperature and extreme weather events such as floods can affect occurrence of generic *E. coli* on fresh produce. 

## 4. Water Treatment Technologies

Leafy greens remain one of the most relevant crops in fresh produce, with an increasing production of bagged salads. However, increased vigilance, in particular related to the selection of water sources and use of water treatment, may help to take safety of fresh produce to the next level. Appropriate irrigation management practices are needed to guarantee the sustainability of the environment and the quality of the leafy greens. The study by Allende and Monaghan [8] provides an overview of the main problems in the production of leafy vegetables associated with irrigation water, including microbial risk and difficulties in water monitoring, compliance with evolving regulations and quality standards, and summarizes the current alternatives available for growers to reduce microbial risks. In addition the Special Issue also includes a dedicated study presenting the data on fecal indicators and selected bacterial pathogens to assess the level of fecal contamination of a Norwegian river used for irrigation in an area which has a high production level of various types of food commodities (Johannessen *et al*. [6]).

The importance of water quality for the rinse step of leafy greens at harvest and also the washing step in the production of fresh-cut produce was identified as a potential pathway for dispersion of spoilage bacteria, fecal indicator bacteria such as *E. coli* or introduction of pathogens via cross-contamination. This was observed, in particular, if no sanitizing agents were used to keep the water clean in the washing tank (Holvoet *et al*.) [9]. Effectiveness of water disinfection needs to be validated case by case, and its appropriate use monitored. The review by Bannach *et al*. [10] examines the efficacy of process wash water disinfectants during produce processing. The conditions for use of chlorine and a range of alternatives such as chlorine dioxide, peroxyacetic acid, ozone in wash water disinfection to prevent cross-contamination were studied. Another study in this Special Issue focused on decontamination of fresh-cut vegetables. Petri *et al*. [11] evaluated the effect of combined decontamination methods based on the use of different sanitizers and the application of pressure on the inactivation of *E. coli* O157:H7 on fresh-cut lettuce and carrots.

## 5. Sampling and Testing

In many studies as mentioned above, sampling and testing has also been noted to be a recurring issue. This is particularly true in the case of emerging pathogens, such as Shiga-toxin producing pathogenic *E. coli* (STEC), *E. coli* O157 being the most dominant serotype within the STEC. Current reference methods for STEC are based upon PCR techniques (ISO/TS 13136, 2012) [12] and have been shown to be technically complex. In addition, information is lacking on the relationship between the detection of the STEC virulence genes by these molecular techniques and the actual presence of the infectious agent and associated risk to public health. There is still the need for actual isolation of both O157 and non-O157 STEC from food samples for further geno-, virulo- and phenotyping, but this has proven to be challenging. Verhaegen *et al*. [13] describes the selection of a suitable selective isolation agar. Six chromogenic agar media were qualitatively and quantitatively evaluated for the isolation of STEC.

Apart from targeted testing to particular micro-organisms, increasingly molecular techniques provide opportunities for non-targeted testing such as the use of a metagenomics approach to study the complexity of the microbiota in foods (Cocolin and Ercolini [14]). The use of advanced molecular techniques may provide added value to study the intrinsic microbial ecology of the host plant which may impact the persistence of pathogens in leafy greens. Ceuppens *et al*. [15] investigated the epiphytic bacterial community of cut basil leaves using culture-independent techniques, including denaturing gradient gel electrophoresis and next-generation sequencing. It was noted that sample preparation had a major influence on the results from the molecular analysis. In addition, spoilage was not associated with specific bacterial groups and presumably caused by physiological tissue deterioration and visual defects, rather than by bacterial growth.

## 6. Vulnerable Consumer Groups and Eating Habits 

Although having elaborated appropriate control measures for ensuring safe food, it has to be acknowledged that microbial and chemical hazards are hard to exclude from the food supply chain and new ones are emerging all the time in particular in times of increasingly sensitive detection methodologies revealing new or lower concentrations of hazards. Even in a well-functioning regulatory and management system one cannot ensure zero risk and the term “zero tolerance” should not be used without knowledge of the scientific and technical factors surrounding it. 

In addition, different consumer groups may have different risk profiles and thus need different guidelines on purchase and food preparation for safe food. Foodborne pathogens are more likely to cause infection and to result in serious consequences in vulnerable people than in healthy adults. Listeriosis is an example of an infectious disease in particular involving immunocompromised persons either at home or residing in elderly homes or hospitals. Although also pregnant women are at risk, in particular the growing elderly and immunocompromised population are a point of attention in the ‘greying’ society in many Western European countries. Often food for these consumer groups is looked at from a dietary point of view and optimized for nutritious purposes improving health. Lund [16] outlined in her review the main factors leading to foodborne disease caused by major pathogens and measures to prevent foodborne disease include procedures based on Hazard Analysis Critical Control Point principles and Pre-Requisite Programs and, especially for vulnerable people, the use of lower-risk foods instead of higher-risk products. Hutchinson *et al*. [17] performed a literature search identifying 40 pâté manufacture recipes. Next they picked and assembled from various recipes the stages with a potential to be antimicrobial to form a new protocol for the preparation of chicken liver pâté and checked by challenge testing that it reliably destroys campylobacters. 

In conclusion, the Special Issue on Food Safety was launched because of an increasing awareness of food safety within a sustainable global food supply. Implementation of food safety management systems alongside the supply chain in different regions needs knowledge from food safety research but also understanding of local practices, context and environmental conditions. In times of increasing concern on food and nutrition security, a debate on stringency of food safety regulation is expected, as strict food safety legislation is sometimes blamed as one of the causes contributing to food waste. This debate is part of the interface of risks assessment, risk management and risk communication. Food safety research will continue to provide insights and is needed to help out in tackling current and emerging food safety challenges in a changing world. 
